# Towards a basic endoscopic evaluation of swallowing in acute stroke – identification of salient findings by the inexperienced examiner

**DOI:** 10.1186/1472-6920-9-13

**Published:** 2009-03-10

**Authors:** Tobias Warnecke, Inga Teismann, Stephan Oelenberg, Christina Hamacher, E Bernd Ringelstein, Wolf R Schäbitz, Rainer Dziewas

**Affiliations:** 1 Department of Neurology, University Hospital of Münster, Albert-Schweitzer-Str. 33, D-48129 Münster, Germany

## Abstract

**Background:**

Dysphagia is common after stroke. Fiberoptic endoscopic evaluation of swallowing (FEES) is a powerful tool for dysphagia assessment. The purpose of this study was to assess whether a previously established endoscopic examination protocol based on the identification of typical findings indicative of stroke – related dysphagia may be learned and adopted by clinicians so far inexperienced in this field.

**Methods:**

After receiving a structured lecture on this topic, participants were asked to rate video sequences of endoscopic swallowing examinations of acute stroke patients. The first part of the testing ("single findings-rating") comprised of 16 single sequences, the second part ("complete examination-rating") presented the key sequences of 8 complete examinations. Before the second part was started, results of the first were discussed.

**Results:**

At the "single findings-rating" 88.8% of video-sequences were assessed correctly, while at the "complete examination-rating" the average performance had improved to 96%. Furthermore, no overlooking of relevant pathologies was noted in the second part of the testing.

**Conclusion:**

This study suggests that the presented endoscopic examination protocol is reliably interpreted by inexperienced clinicians after a short lecture and may therefore easily and successfully be adopted in dysphagia management of acute stroke care.

## Background

A proper assessment of dysphagia is one of the most important topics in acute stroke care. Although clinical dysphagia screening is known to reduce the risk of pneumonia and fatal outcome after stroke [1-3], this approach has an insufficient sensitivity, specificity and interrater reliability, and is particularly poor in detecting silent aspiration [4,5]. First evidence is evolving that fiberoptic endoscopic evaluation of swallowing (FEES) may be particularly helpful in this context [6,7]. This procedure can be performed at the bedside like clinical examination and is generally well tolerated and therefore frequently repeatable [6,8]. According to recent studies, FEES performs even better than videofluoroscopy in detecting important consequences of dysphagia, in particular aspiration and pharyngeal residue severity [9,10].

Recently, we developed a FEES-based dysphagia score that allows a quick deduction of clinical consequences from easy-to-identify endoscopic findings and may therefore efficiently guide management of stroke-related dysphagia in the acute stage of the illness [4].

FEES is usually considered to be a technique that needs a lot of experience to identify and interpret its findings. In the present study, we investigated whether our FEES-based dysphagia score for acute stroke patients may be adopted successfully after a short training session by physicians so far inexperienced in the field of endoscopic dysphagia assessment.

## Methods

### Dysphagia assessment

**Figure 1:**
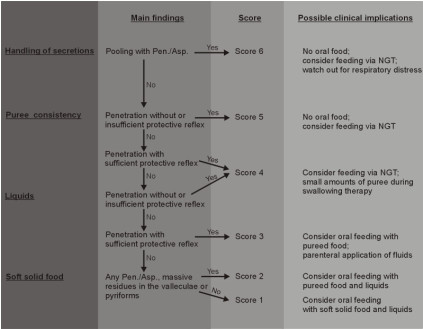
**Flowchart for the endoscopic assessment of dysphagia**.

Previously, we developed a protocol for endoscopic assessment of dysphagia in patients with acute stroke. Following this protocol each examined patient is classified according to a six point scoring system that allows a quick deduction of clinical consequences (Figure 1) [4,11]. In brief, the examination starts with rating the severity of oropharyngeal secretions (handling of secretions). In case saliva pooling with penetration or aspiration is found severe dysphagia is suspected and score 6 is given. Patients being able to handle their saliva without penetration or aspiration and do not have a severely reduced state of consciousness (i.e. stupor or coma) receive a teaspoon of puree consistency next. Those who show penetration or aspiration without protective reflex (i.e. coughing or swallowing) on at least one of three attempts are again diagnosed with severe dysphagia (score 5). If sufficient protective reflexes are present score 4 is attributed.

Patients managing puree consistency are exposed to a teaspoon of colored water. Penetration or aspiration without sufficient protective reflex keeps the patient on score 4, while the presence of protective reflexes leads to score 3.

If patients being able to swallow liquids three times without penetration or aspiration, a small piece of white bread is given to them at the last step. Here, penetration or aspiration or severe residues (> 50% of bolus size, i.e. bolus fills or overflows the cavities) in the valleculae or pyriforms are taken as evidence of severe difficulty with this food consistency resulting in score 2. If none of these findings are observed on three consecutive trials score 1 is given.

### Subjects

Seventeen neurologists (7 women, 10 men; mean age = 31 years, range 28 to 45 years) with a professional experience of 4.5 years (range 1–16 years) took part in the study. None of the participants had experience with fiberoptic endoscopic dysphagia assessment.

### Lecture

First, a 30-minutes lecture on the physiology of swallowing and the basics of FEES was given. The above mentioned salient findings were defined, the dysphagia assessment protocol was explained and characteristic videos were shown.

### Evaluation-videos and test construction

Suitable video sequences of endoscopic swallowing examinations of acute stroke patients with duration of 5 to 10 seconds were collected by one of the authors (S.O.) from archived material. Two raters (R.D., T.W.), both having several years of experience with this diagnostic procedure, separately evaluated the salient findings of each sequence. 5% of the collected samples were rejected due to disagreement between the two raters.

The first part of testing ("single findings-rating") comprised of 16 sequences presented to the inexperienced raters in succession without intermediate explanation or discussion. On request, sequences were repeatedly shown. Four samples each belonged to the category "handling of secretion", "puree consistency", "liquid", and "semisolid food", respectively. The raters were asked to evaluate the videos for the presence or absence of penetration or aspiration on a 5-point scale ("No penetration or aspiration", "Penetration with protective reflex", "Penetration without protective reflex", "Aspiration with protective reflex", and "Aspiration without protective reflex"). Additionally they had to assess severity ("No", "Mild", "Moderate", "Severe") and location ("No", "Valleculae", "Sinus piriformis") of residues that were left after the swallow [9]. After finishing the evaluation of the 16 video sequences by the inexperienced raters, all scoring sheets of the first part of testing were collected. Subsequently, the videos were briefly reviewed and evaluations were discussed. In this way, apart from serving as test instrument, the single findings-rating was also used as interactive teaching tool.

During the second part of testing ("complete examination-rating") the key sequences of 8 complete FEES examinations were presented. Three patients with infarction of the left middle cerebral artery (MCA), three with infarction of the right MCA, and two with brainstem infarction were chosen. According to the protocol described above, after each sequence, the raters had to state whether they wanted to finish the examination and decide on a score, because, for example, penetration was detected, or whether the examination should proceed to the next food consistency. Rating was done silently on a prepared score sheet.

### Statistics

For each task, the percentage of correct ratings was determined. Furthermore, inter-rater reliability was assessed by calculating kappa (κ) coefficients. The upper limit of the κ-coefficient representing total agreement between the raters is 1.0, while a κ-coefficient of 0.0 represents agreement at chance level. κ-coefficient scores in the range of 0.41 to 0.60 indicate moderate agreement, 0.61 to 0.80 indicate substantial agreement, and 0.81 to 1.00 indicate excellent agreement [12].

## Results

All participants completed the study. As is shown in table 1, at the "single findings-rating" 88.8% of video-sequences were assessed correctly. The corresponding κ-coefficient of 0.73 indicated substantial agreement between the raters (table 2).

**Table 1:** Results of the "single findings-rating": Percentage of correct ratings

	**Task Number**	**Main finding in the video sample**	**Correct ratings**
**Handling of Secr.**	1	Aspiration without protective reflex	94%
	2	No penetration or aspiration	100%
	3	Aspiration without protective reflex	100%
	4	Penetration with protective reflex	94%

	Mean Score		**97%**

**Puree consistency**	5	No penetration or aspiration; no residues	76%
	6	Penetration without protective reflex; no residues	100%
	7	Aspiration without protective reflex; no residues	82%
	8	Aspiration without protective reflex; residues in s.p.	76%

	Mean Score		**83.5%**

**Liquids**	9	No penetration or aspiration	82%
	10	Penetration with protective reflex	53%
	11	No penetration or aspiration	100%
	12	Aspiration with protective reflex	100%

	Mean Score		**83.8%**

**Semisolid food**	13	No penetration or aspiration	94%
	14	Penetration without protective reflex; residues in the vallec.	100%
	15	Penetration without protective reflex; residues in the s.p.	76%
	16	Penetration without protective reflex; residues in the vallec.	94%

	Mean Score		**91%**

**Overall**	Mean Score		**88.8%**

Secr. = Secretions, s.p. = sinus pyriformis; vallec. = valleculae.

This table summarizes the percentage of correct ratings in the "single findings-rating". Four video sequences (second column) belong to the category "handling of secretion", "puree consistency", "liquids", and "semisolid food", respectively. The third column gives the main finding of the respective video sample. In the fourth column the percentage of correct ratings is stated. For example, the third video sequence of "puree consistency" showed as main pathological finding "aspiration without protective reflexes, no residues". 82% of the raters suggested the correct finding.

**Table 2:** Results of the "single findings-rating": κ-coefficients

**Video categories**	**κ-coefficients**	**p**
**Handling of Secr.**	0.91	< 0.001
**Puree consistency**	0.60	< 0.001
**Liquids**	0.63	< 0.001
**Semisolid food**	0.66	< 0.001
**Overall**	0.73	< 0.001

Secr. = Secretions

This table summarizes the corresponding κ-coefficients for each sample of different video categories in the "single findings-rating".

With over 90% of correct ratings the items "handling of secretions" and "semisolid food" achieved the best results, while "liquids" and "pureed food" were still rated correctly in nearly 84% of cases. The corresponding κ-coefficients ranged from 0.60 (puree consistency) to 0.91 (handling of secretions) (table 2). Looking at different types of salient findings, assessing penetration proved to be relatively difficult with 14% mistaken judgments. Aspiration and normal findings were rated better, wrong assessments occurred in only 9% of cases.

At the "complete examination rating" the participants' diagnostic abilities had improved. Altogether, only five mistakes occurred, giving an average of 96% of correct scores and a corresponding κ-coefficient of 0.91 (p < 0.001) indicating excellent agreement between the raters (Table 3).

**Table 3:** Results of the "complete examination-rating"

	**1**	**2**	**3**	**4**	**5**	**6**	**7**	**8**
Clinical situation	72 yr, male, MCA right, NIH-SS 8, mild f.p., dysarthria, exam. at day 2	77 yr, female, MCA right, NIH-SS 7, moderate f. p.; exam. at day 1	67 yr, female, MCA left, NIH-SS 10, buccofac. apraxia, exam. at day 1	71 yr, male, MCA left, NIH-SS 12, severe f.p.; exam. at day 2	78 yr, male, BS, NIH-SS 7, severe dysarthria; exam. at day 1	74 yr, male, MCA left, NIH-SS 9, severe f.p., dysarthria; exam. at day 1	63 yr, male, BS, NIH-SS 8, severe dysarthria; exam. at day 1	72 yr, female, MCA right, NIH-SS 8, moderate f.p., neg-lect; exam. at day 1

Handling of Secretions	No pen./asp.	No pen./asp.; Saliva in the s.p.	No pen./asp.; Saliva in the s.p.	No pen./asp.; Saliva in the s.p	No pen./asp.	No pen./asp.	Asp. without prot. reflex	No pen./asp.

**Decision**	proceed	proceed	proceed	proceed	proceed	proceed	Score 6	proceed

**Correct rat.**	100%	100%	100%	94%	100%	94%	100%	100%

Puree consistency	No pen./asp., residues in the s.p.	No pen./asp.	No pen./asp.; residues in the s.p.	Pen. without prot. refl.	Pen. with prot. refl.	Pen. without prot. refl.	n.a.	No pen./asp.; residues in the s.p.

**Decision**	proceed	proceed	proceed	Score 5	Score 4	Score 5	n.a.	proceed

**Correct rat.**	100%	100%	94%	100%	100%	100%	n.a.	88%

Liquids	No pen./asp.	No pen./apsp; Leaking s.p.	Pen. with prot. reflex	n.a.	n.a.	n.a.	n.a.	Asp. without prot. reflex

**Decision**	proceed	proceed	Score 3	n.a.	n.a.	n.a.	n.a.	Score 4

**Correct rat.**	100%	100%	100%	n.a.	n.a.	n.a.	n.a.	100%

Semisolid food	Severe residues in the vallec.	No pen./asp.; few residues in the vallec.	n.a.	n.a.	n.a.	n.a.	n.a.	n.a.

**Decision**	Score 2	Score 1	n.a.	n.a.	n.a.	n.a.	n.a.	n.a.

**Correct rat.**	100%	100%	n.a.	n.a.	n.a.	n.a.	n.a.	n.a.

Correct score	100%	100%	94%	94%	100%	94%	100%	88%

Correct rat. = Correct ratings; f.p. = facial palsy; buccofac. = buccofacial; somnol. = somnolence; s.p. = sinus pyriformis; vallec. = valleculae; MCA = stroke in the territory of the middle cerebral artery; BS = brainstem stroke; NIH-SS = National Institute of Health Stroke Scale Score. The video samples were rated according to the screening protocol outlined in figure 1.

Interestingly, no instance of overlooking a relevant pathology was noted, whereas all wrong assessments belonged to the category "false positive" rating. In three cases, residues in the sinus pyriformis were misleadingly taken as reason to "terminate" the examination by suggesting a score, while twice saliva pooling in the sinus pyriformis without penetration or aspiration provoked an overly cautious approach.

## Discussion

This study demonstrates that in acute stroke patients assessing salient findings of endoscopic swallowing examination following the here described protocol can be done reliably with a minimum of experience. After receiving an introductory lecture the previously untrained participants of our study gave a correct rating of characteristic video sequences in nearly 90% of cases, which corresponded to a κ-coefficient of 0.73.

The assessment of liquid and pureed food swallows proved relatively difficult in comparison to semisolid food. This is probably because the former consistencies typically showed predeglutitive key findings only for fractions of a second. Here, replaying of the respective sequences in slow motion, which is also advocated by Langmore and co-workers [13], might have been useful. The further improvement seen at the complete examination-rating with 96% correct assessments (corresponding κ-coefficient = 0.91) was probably due to a marked training effect of the previous testing and the subsequent discussion. Since systematic curricula on the present subject are sparse, this point may be of relevance for future teaching of endoscopic evaluation of swallowing.

Looking in detail at the few mistakes made at the second testing, it is striking that in each single case participants erred on "safe side" leading to a worse score than necessary.

Furthermore, the excellent κ-coefficient achieved by the previously untrained participants of our study at the complete examination-rating may suggest that endoscopic swallowing assessment according to the here described protocol is simpler to score and more reliable compared to videofluoroscopic swallowing evaluations of acute stroke patients [5,14].

When we developed our screening protocol for acute stroke patients, we aimed for a simple way of scoring endoscopic key findings making this tool more easy to adopt for inexperienced clinicians [4]. Thus, in comparison to the well-known 8-point Penetration-Aspiration Scale (PAS) developed by Rosenbek and co-workers [15], we chose a simplified 5-point scale to score penetration and aspiration events. By using this 5-point scale, the here described endoscopic dysphagia screening protocol showed an excellent interrater reliability when applied to acute stroke patients [4].

Apart from being able to correctly identify key findings of endoscopic swallowing examination, our protocol for acute stroke patients also requires demanding technical skills from the clinicians. Murray suggests that, after observing several examinations, the novice should perform at least 20 to 30 examinations under supervision before starting to work on his own [13]. Therefore, when intending to involve so far inexperienced clinicians in the endoscopic examination of swallowing, one has to provide sufficient practical instructions along with the mentioned theoretical tutorials.

Moreover, we would like to emphasise that the inexperienced clinicians participating in our study did not score and interpret complete FEES examinations, but only a small number of relevant parameters for the purpose of dysphagia screening. In comparison to the examination protocol outlined here, Langmore's original FEES protocol is clearly more differentiated [16]. Apart from identifying salient findings, it also comprises of additional important aspects like a detailed anatomic-physiologic assessment including a pharyngo-laryngeal sensory testing [8,17], evaluation of therapeutic maneuvers and their effect on the swallow, and testing of different methods of food delivery. Furthermore, the interpretation of endoscopic findings is not confined to the assessment of the aspiration risk alone but deduces the underlying pathophysiology of dysphagia for suggesting suitable therapeutic interventions and for giving a prognosis. Of course, analyzing and interpreting abnormal findings according to this differentiated and comprehensive protocol needs a lot more of clinical experience with the FEES procedure that cannot be learned in a 30 minutes-lecture.

## Conclusion

Our study results give evidence that key findings of endoscopic swallowing evaluations of acute stroke patients and the deduction of clinical consequences can reliably be identified by so far inexperienced physicians after receiving a short and structured teaching. Therefore, we believe that the present approach will help FEES to be easily and successfully adopted in dysphagia management of acute stroke care.

## Abbreviations

FEES: Fiberoptic endoscopic evaluation of swallowing; MCA: Middle cerebral artery.

## Competing interests

The authors declare that they have no competing interests.

## Authors' contributions

TW, IT, SO, CH and RD were involved in the FEES. TW, EBR, WRS and RD designed the study protocol. TW and RD performed statistical analysis. TW wrote the manuscript. WRS and EBR read previous drafts of the manuscript and made substantial improvements. All authors read and approved the final manuscript.

## Pre-publication history

The pre-publication history for this paper can be accessed here:


